# Tracing microplastics in aquatic environments based on sediment analogies

**DOI:** 10.1038/s41598-019-50508-2

**Published:** 2019-10-23

**Authors:** Kristina Enders, Andrea Käppler, Oliver Biniasch, Peter Feldens, Nicole Stollberg, Xaver Lange, Dieter Fischer, Klaus-Jochen Eichhorn, Falk Pollehne, Sonja Oberbeckmann, Matthias Labrenz

**Affiliations:** 1 Leibniz Institute for Baltic Sea Research Warnemünde (IOW), Seestraße 15, 18119 Rostock, DE Germany; 2 Leibniz Institute for Polymer Research Dresden (IPF), Hohe Str. 6, 01069 Dresden, DE Germany

**Keywords:** Environmental impact, Physical oceanography

## Abstract

Microplastics (MP) data collection from the aquatic environment is a challenging endeavour that sets apparent limitations to regional and global MP quantification. Expensive data collection causes small sample sizes and oftentimes existing data sets are compared without accounting for natural variability due to hydrodynamic processes governing the distribution of particles. In Warnow estuarine sediments (Germany) we found significant correlations between high-density polymer size fractions (≥500 µm) and sediment grain size. Among potential predictor variables (source and environmental terms) sediment grain size was the critical proxy for MP abundance. The MP sediment relationship can be explained by the force necessary to start particle transport: at the same level of fluid motion, transported sediment grains and MP particles are offset in size by one to two orders of magnitude. Determining grain-size corrected MP abundances by fractionated granulometric normalisation is recommended as a basis for future MP projections and identification of sinks and sources.

## Introduction

The majority of the plastic that reaches aquatic environment originates from land-based sources^1^ and is thought to eventually reach the sea via streams and rivers, with sea basins as an ultimate sink for particles^2,3^. This concept is widely accepted in scientific literature and leads to the preclusion of microplastics (MP) deposition and temporary or long-term storage in rivers and coastal environments and thus potentially results in an underestimation of the total plastic removal. Recent studies (e.g.^4,5^) showed the potential of freshwater systems to retain a substantial fraction of MP. As deposition is the dominant sink for MP in aquatic environments^6,7^ it is important that the controlling mechanisms are studied and understood^8–10^.

A valid estimation of the actual plastic load in sediments is currently prevented due to the lack of data analysis standards that account for natural variability and the generally low sample densities. The observation that small scale spatial variations in MP abundance can exceed those across larger spatial scales^10^ indicates that local hydrodynamic conditions that influence particle motion have to first be taken into account before valid comparisons across temporal and spatial scales can be made. Being part of the general pool of suspended solids within a specific hydrodynamic regime, the distribution dynamics of MP - likewise all natural particulate matter – can be obtained from the physics of particle motion. With decreasing sediment grain sizes the threshold for the initiation of particle motion decreases^11^. Thus, the sediment grain size distribution is mainly determined by the hydrodynamic (turbulence) regime; for non-cohesive particles, fine-grained sediments are found in low-energy environments, coarser sediments in high-energy environments^12^. Accordingly, a relationship between organic (particulate) matter and sediment grain size has often been described^13^.

Hereby, MP correlations to ubiquitous and natural particles, such as organic matter or siliclastic sediments could be useful tools to infer MP contamination levels. In other contaminant studies granulometric and geochemical normalisation approaches are standard^12^. Herein, normalisation is defined as a mathematical procedure to adjust MP abundance values for the influence of the natural variability in sediment granulometry induced by the energetic condition of the environment. Comprehensive surveys continue to be expensive due to the wide variety of synthetic polymer composites collected under the umbrella term ‘microplastics’ requiring extensive sample preparation measures. The development of proxies for MP data would allow for sensible extrapolations to larger spatial scales which is ultimately necessary for a quantification of the MP contamination in aquatic systems across the world.

Only relatively few studies have systematically examined sedimented MP in relation to environmental conditions that influence particle transport. Conclusions on whether or not MP abundance varies with sediment composition, such as grain size or organic matter content, were vastly diverging. Whereas Strand *et al*., Maes *et al*. and Vianello *et al*.^14–16^ found indications for the existence of such a relationship, others^9,17–22^ could not confirm it. Such controversy in the literature exemplifies the existing knowledge gaps for a potential MP - sediment relationship. In this study, it is hypothesised that MP distribution patterns can be approximated by patterns of sedimentary composition. This relationship is investigated in sediments of an estuary that receives freshwater from the Warnow river in Germany and flows to the Baltic Sea. The selected site is representative of a large sediment grain size distribution and composition and an expectantly significant exposure to MP contamination due to intensive anthropogenic usage (urban, industrial).

The overall aim of this study is to establish the basis for a proxy for rational MP distribution maps which account for heterogeneous sedimentary environments. At first a conventional documentation of MP abundances and composition and a characterisation of MP intrinsic physical properties with respect to spatial distribution is provided for the area studied. In order to explore the parameters that determine the distribution of MP in the Warnow estuary generalised linear models (GLM) were developed. This multiple regression analysis was performed on a large set of potential MP sources (e.g. population density, marinas, etc.) and environmental parameters (e.g. sediment grain size, depth, etc.). Major factors were then analysed in detail. As a result the principal part of the study focuses on the MP-Sediment relationship. A correlation analysis is performed between MP fractions, defined by physical properties, and sediment grain size fractions. The empirically derived relationships are evaluated based on fundamental sediment transport concepts (critical shear stress^11^). Limitations of the analysis are being discussed. For validation of the results with respect to the hydrodynamic conditions in the Warnow estuary shear stress data was retrieved from an established hydrodynamic coastal ocean model^23^. By compiling available MP data sets from the literature that provide sediment grain size parameters, major influential factors on the quality of the MP - sediment relationship were identified. Exemplary offshore data (deep basins from the Baltic Sea) is provided for comparison based on the presented integrated normalisation approach. Additional one-year sediment trap samples complemented the analysis in order to improve our understanding of long-distance MP transport and sea-based sources. Finally, land-based point sources and recommendations and prospect of the granulometric normalisation approach of MP data is summarised.

## Results and Discussion

### Initial analysis

#### MP abundance and composition in the Warnow estuary

**Figure 1:**
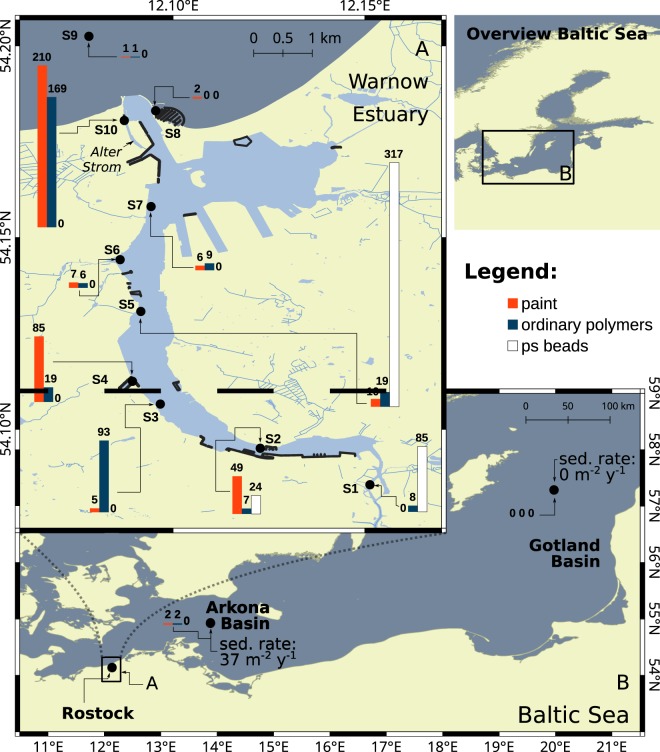
MP abundance maps of all sediment samples taken: (**A**) the Warnow estuary and **(B)** Arkona and Gotland basins in the Baltic Sea (referred section: MP contamination levels comparison to Baltic Sea basins). Sedimentation rates derived from sediment trap sampling in the two basins are displayed next to the stations. Blue bars indicate the occurrence of ordinary polymers, red bars paint resins and white bars PS beads. Respective abundances [kg^−1^ DW] are shown above the bars. Representative replicates (n = 3) were available for station S2 and S10 amounting to 78 ± 10 kg^−1^ DW and 379 ± 28 kg^−1^ DW, respectively. Possible deviations when adding up polymer type-specific abundances can arise due to rounding differences. Marinas and harbours are shown as black shapes. Sampling stations are highlighted as black circles along with the respective station number. Presented maps are projected in a geodetic system WGS 84 (EPSG:4326) and were created in QGIS^62^ using OpenStreetMaps (OSM) data^64^.

Total MP (TMP) abundances along the river bed were highest in the upper part of the estuary and then steadily decreased downstream towards the mouth of the river (Fig. 1A). Abundances appear to range over two orders of magnitude and assigned to three different areas. The upper Warnow estuary (S1–S7) showed a median TMP abundance of 93 [46–100] kg^−1^ dry weight (DW), with one station standing out with higher relative MP numbers of 346 kg^−1^ DW (S5). The Baltic Sea opening had comparatively low abundances of 2[2-2] kg^−1^ DW (S8, S9), whereas the Alter Strom (adjacent side arm of the Warnow estuary) revealed highest levels of 379 ± 28  kg^−1^ DW (n = 3, S10) (Fig. 1A) of the area studied.

MP were categorised based on polymer types and morphology. That is, ordinary polymers (Fig. 1A, blue bars) including those of higher density (HD) and those of lower density (LD) than water (*ρ*_*Warnow*_ = 1.00–1.01 *g cm*^−3^), paint resins (Fig. 1A, red bars) and micro polystyrene (PS) beads (Fig. 1A, white bars). Most of these PS beads were spectroscopically identified as a variety of ion exchange polymers and dominated TMP by 58% in the estuary. Specifications of the identified PS beads are provided in the supplementary information (SI), Fig. S1. In total, paint and ordinary polymers were equally common across the sample set. A more detailed description for the composition and distribution of each MP category is provided in the SI, Text S1.

#### Fractionation of MP species based on physical properties

The diversity of plastic compositions brings with it a variety of properties that influence particle transport hampering the prediction of TMP distribution based on one single proxy. In order to identify MP distribution patterns within the present hydrological environment MP were grouped according to the most determining intrinsic variables with regard to particle transport behaviour: size, density and shape^11,24^.

*Size*: In the estuary, the size distribution appeared to be dependant on density with significant differences between HD and LD polymers (SI, Fig. S2A). HD polymers increased in numbers with smaller sizes, following a power law regression scaling with an exponent of −3.88 (Spearman: *r*_*s*_ = − 1, p = 0.08). Whereas, LD polymers remained at a constant low level without variations in size. As an exception, one sample is positioned in a side arm (Alter Strom) of the Warnow estuary which connect at the estuary mouth. Here, both LD and HD polymers show a decrease in abundance below 1000 *μ*m (SI, Fig. S2B).

*Density*: Spectroscopically identified polymer types were used to infer typical density ranges. Ordinary polymers composed the group of poleolefines, including polypropylene (PP) and polyethylene (PE), acetates, including copolymeric polyvinylacetate and ethylenvinylacetate (PVAc/EVA), polystyrenes (PS), polyamides (PA), acrylates, including polymethylmethacrylate (PMMA) and polyacrylonitrile (PAN), and polyvinylchlorides (PVC). These polymers span a large range of typical densities from 0.89–1.41 g cm^−3^ ^25^, summarised in^26^. Excluded from this analysis were polytetrafluorethylene (PTFE) and polyethylene terephthalate (PET), as they appeared in contamination controls (SI, Text S2). Based on a report from the EPA^27^ we calculated that paint resins possess an average density of approximately 1.6 g cm^−3^. PS beads were density categorised according to their basis polymer PS (see SI, Text S1).

**Figure 2:**
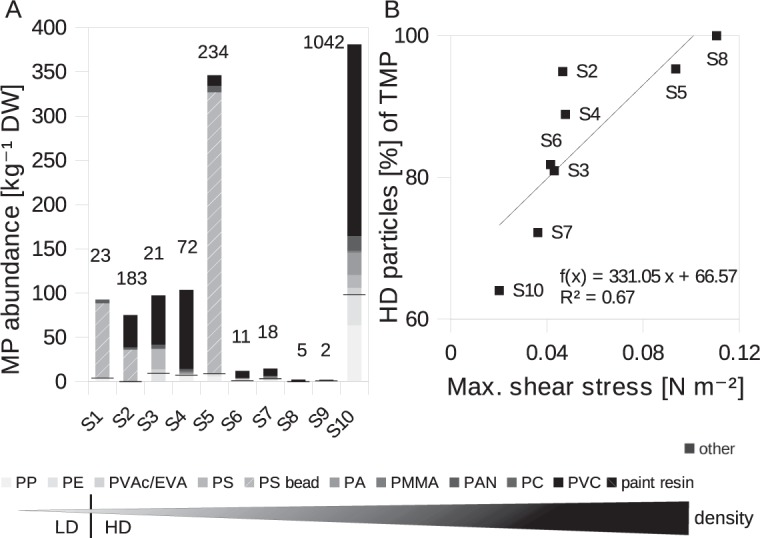
MP distribution by polymer type with distinct given densities and their dependency of shear stress. (**A**) MP distribution with numbers n above the bars. Grey scaling indicates the corresponding given densities of the polymers, increasing from light to dark (0.89–1.6 g cm^−3^). The black line indicates the transition from LD to HD polymers determined by the density of water (~1 g cm^−3^). The polymeric category “other” consists of a variety of different HD polymers and consequently displayed separately from the grey scale. **(B)** HD particles (= TMP - LD - HD fibres) percentage of all polymers as a function of simulated maximum shear stress in the Warnow estuary. Two stations were excluded from the correlation for reasons of missing coverage by the hydrodynamic model (S1) or low sample size (S9).

HD polymers were more than twice as abundant as LD polymers within the ordinary polymer category along the estuary. By density, paint and PS beads were also assigned to the category of HD polymers which resulted in a percentage contribution of approximately 90% and 95% within the overall data set and Warnow estuary (excl. Alter Strom), respectively. The plastic composition is, thus, clearly dominated by HD polymers (Fig. 2A). In the Alter Strom sediments, LD polymers were more abundant, with 23%. Otherwise, the overall polymeric distribution largely coincided with those found in the estuary.

The critical shear stress, calculated for the Warnow estuary stations (simulated maximum over one year, see section Hydrodynamic model), is introduced as an important measure of the energetic environment (Fig. 2B)^11^. Generally, the larger the shear stress forces on the sediment bed the larger the proportion of HD polymers (r = 0.73, p = 0.04) relative to LD polymers (HD = TMP − LD). The significance level of this correlation rose (r = 0.82, p = 0.01) when excluding HD fibres, hence only HD particulate polymers were considered (Fig. 2B). The influence of shape is addressed in the following paragraph. Conclusively, hydrodynamic conditions appear to be an important influential factor on the selection of different polymer types i.e. densities.

*Shape*: Shape categories were split into fibres and particles, the latter of which was dominated by irregular fragments and spheres. Fibrous MP were usually composed of PAN, PA and PP and remained at a rather low level in the estuarine transect. In the Alter Strom, fibres reached abundances of 84 ± 45 kg^−1^ DW, roughly equalling particulate ordinary polymers. Although most fibres by density can be related to the HD polymer fraction (64%, overall), their rather dispersed distribution pattern aligned much better with that of non-fibrous LD polymers (r = 0.99, p < 0.001).

#### Exploration of determining parameters for MP distribution

GLMs were developed to explore which parameters - potential sources and/or environmental factors - can explain MP distribution patterns in the Warnow estuary. The abundance of each MP category, paint resins, ordinary polymers, PS beads (restricted to ion exchangers) as well as TMP was analysed separately in order to find type specific emitters. Tested potential source terms were: distance to closest waste water treatment plant (WWTP), marina, recycling station, mixed water and rain water sewers as well as population density, number of tourism activity points, industrial areas and metal companies in 1000 m radius. Environmental parameters included physical parameters such as salinity, depth and maximum shear stress. Further, different types of natural (particulate) matter such as organic matter (total organic carbon (TOC) × 2.22), TOC/N, sediment grain size (<63 *μ*m) and CaCO_3_ were tested to evaluate their potential usage as proxies. This is based on the assumption that various types of particulate matter and MP are similarly influenced by the present hydrodynamic conditions. A table including values of explanatory variables with units, parameter definition and references of data acquirement is included in the SI, Table S3.

**Figure 3:**
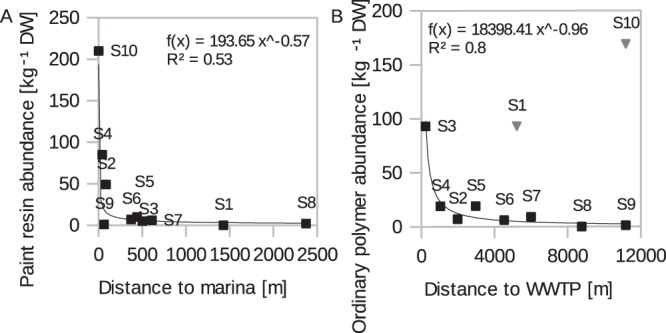
Relationship of MP and major identified sources: (**A**) between paint resin abundance and distance to marinas per station (n = 10) and **(B)** ordinary polymers to distance to WWTP per station (n = 8). The outliers S10 and S1 (grey triangles) would decrease the regression fit r^2^ to 0.09 and were consequently excluded from the analysis.

Explanatory variables (unstandardised) represent an estimation of parameter influence in original units and are given in descending order of importance. This is evaluated by the change of several statistical parameters such as decrease of deviance, Akaike information criterion (AIC), increase in coefficient of determination *r*^2^, level of significance, t-values and residual analysis. All predictor terms that composed the final models attained a level of significance of p < 0.05. Further GLM specifications and residual analysis are provided in the Materials and Methods section and SI, Fig. S3A–C. Best model fits for the individual MP categories are composed as follows:

1 $$\log (\widehat{{\rm{paint}}\,{\rm{resin}}})=2.352+0.032\,{\rm{Grain}}\,{\rm{Size}}\,(\, < \,63\,\mu m)-0.003\,{\rm{Distance}}\,{\rm{Marina}}$$

with *r*^2^ = 0.97, a residual deviance of 54 with 7 degrees of freedom and Chi-squared vs. constant model: 725, p < 0.001. The strongest impact (r^2^ = 0.84, p < 0.001) on the model for paint resins (Eq. (1)) had the term sediment grain size (<63 *μ*m). Additionally, the distance to marinas (and harbours), one potential source term, showed significant impact. In other words, the MP composition of stations close to marinas and harbours were dominated by paint resins. This significant correlation is even supported when it is used as the only predictor (Fig. 3A, Spearman: *r*_*s*_ = − 0.7, p = 0.03). Industrial areas, including metal and ship constructing sectors also fit paint resin distribution, however, had to be excluded due to model overfitting.

2 $$\log (\widehat{{\rm{ord}}{\rm{.}}\,{\rm{polymer}}})={\rm{0.505}}+{\rm{0.0452}}\,{\rm{Grain}}\,{\rm{Size}}\,(\, < \,{\rm{63}}\,\mu m)+{\rm{0.0249}}\,{\rm{Org}}{\rm{.}}\,{\rm{Matter}}$$

with *r*^2^ = 0.77, a residual deviance of 199.1 with 7 degrees of freedom and Chi-squared vs. constant model: 431, p < 0.001. Apart from sediment grain size distribution, organic matter had an explanatory power for ordinary polymers (Eq. (2)). A reason for this could be the overlap in density ranges, contrarily to the heavier paint resins. Initially, the distance to the WWTP was the most significant source term for local ordinary polymer abundance. However, the occurrence of two outliers and consequent model overfitting excluded this term from the final model. When excluding the outliers the model fit improved (*r*^2^ = 0.92, among other parameters of evaluation) now containing both grain size (<63 *μm*) and distance to WWTP as significant explanatory variables. This indicates that distance to WWTP ascribes a type specific emitter. The linear regression between ordinary polymers and the distance to WWTP is presented separately (Spearman: *r*_*s*_ = −0.84, p = 0.01, Fig. 3B). It is noted that PS beads from station S1 were added to the pool of ordinary polymers as they were not identified as ion exchangers (Fig. S1) and likely originate from different sources. They were not explained by the distance to the WWTP either (see outlier S1, Fig. 3B) and possibly diverge due to the increased distance of S1 upstream of the WWTP. Station S10 is furthest away from the WWTP and situated in a sheltered side arm of the Warnow wherefore additional source terms or deviating hydrodynamic conditions could explain the divergence from the regression fit.

Available source terms could not explain the distribution pattern of ion exchanger beads. This may be due to the versatile applicability of these beads. At the same time they could originate from a specific point emitter which might not be covered by the identified industrial activities used for the parameterisation.

3 $$\begin{array}{c}\log (\widehat{{\rm{TMP}}})=-\,{\rm{0.4207}}+0.0826\,{\rm{Grain}}\,{\rm{Size}}\,(\, < \,{\rm{63}}\,\mu m)+{\rm{0.056}}\,{{\rm{CaCO}}}_{{\rm{3}}}\\ \,\,\,\,\,\,-{\rm{0.0002}}\,{\rm{Distance}}\,{\rm{WWTP}}\end{array}$$

with *r*^2^ = 0.99, a residual deviance of 20.7 with 6 degrees of freedom and a Chi-squared vs. constant model: 1280, p < 0.001. TMP distribution (3) was best explained (*r*^2^ = 0.58) by the fine fraction of sediments (<63 *μ*m). The correlation with CaCO_3_ is introduced based on a cooccurrence with ion exchangers. Apart from a possible correlation in transport behaviour with CaCO_3_ (if e.g. from fragmented mussel shells), it could also be indicative of the composition of the ion exchanger polymers or related application processes (i.e. decalcification) which would lead to increased CaCO_3_ levels. Regarding the small set of data points statistical robustness and causality of this correlation is not clear. If ion exchargers were excluded from TMP the relative importance of sediment grain size increased (*r*^2^ = 0.95).

Conclusively, the MP composition found in the Warnow sediments can be explained by both the co-occurrence of natural (particulate) matter and potential source terms. Local occurrences of paint resins and HD ordinary polymers could, in turn be used as specific source indicators. Diffuse sources were generally difficult to fit as the impact is more dispersed and indiscernible from unexplained statistical deviations or unexplored source terms. A higher resolution and larger sample size could likely result in higher model accuracy. Independent of the MP categories, sediment grain size (<63 *μ*m) appeared as the most prominent explanatory parameter. This suggests, that both materials are similarly influenced by present hydrodynamic conditions. Lower energetic environments would have the capacity to trap more MP of averagely smaller sizes compared to higher energetic environments. The selected fine sediment fraction (<63 *μ*m) is the most widespread granulomeric normaliser of contaminants in use. This specific size threshold is principally based on physical properties (point at equal particle bond - weight ratio) and a measure of the clay fraction characterised by a high binding capacity and coating formation e.g. with organic matter (reviewed in^12^). However, the variable nature of grain size spectra is reduced to only one parameter at the expense of accuracy. Therefore sediment grain size should undergo a more detailed evaluation with respect to their potential usage as a proxy for MP contamination levels.

### MP - Sediment relationship

#### Field data

**Figure 4:**
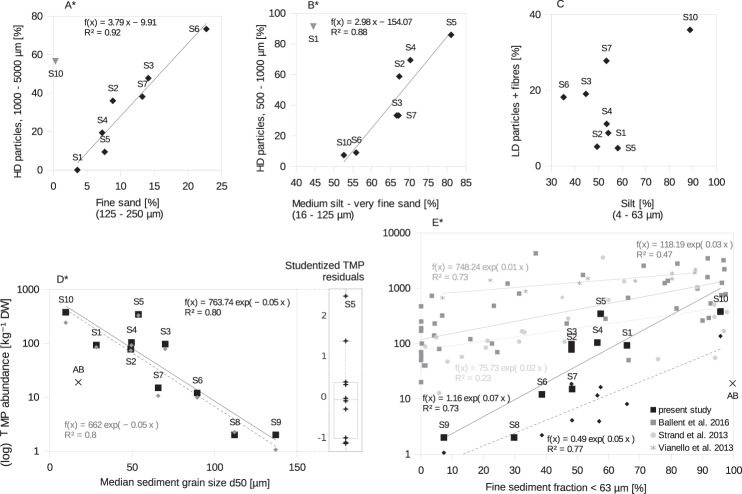
Relationships between MP and sediment distribution patterns. (**A**–**C**) Correlation analysis of specific MP and sediment grain size fractions in relative abundances. The inclusion of the outliers (shown as grey triangles) S10 and S1, would unduly influence the regression fit (decrease of r^2^ to 0.31 and 0.07, respectively) and were consequently excluded from the analysis. **(D)** Correlation of TMP (log transformed, black squares) and HD polymers (grey diamonds with dashed line) as absolute values with median grain size (d50). **(D**, insert) Residual analysis of TMP values normalised by the median grain size (d50). Individual studentised residuals are shown as pluses non of which is deemed a clear outlier. **(E)** Fine sediment fraction (<63 *μm*) against TMP abundance (log transformed, black squares). Y-axis label is shared with **(D)**. Graphs in different shades of grey present an assemblage of available study sites^9,14,16^ showing comparable relationships. Small black diamonds with the dashed line represent LD polymers and fibres. For later reference, in both **(D)** and **(E)**, the respective position of the Baltic Sea sample, Arkona Basin (AB, d50 = 17.3 ± 1.7 *μ*m, <63 *μ*m = 99.6 ± 0.4%, n = 4 ^36^), is marked as a cross. The Gotland Basin with comparable sediment grain size features (GB, <63 *μ*m = 95.7 ± 3.5%, n = 3 ^65^) does not display in the log rendering as MP numbers equal zero. The used grain size data for the two Baltic Sea sediment samples showed neglectable variation across our sampling region^36,65^. Significant correlations are marked with asterisks. All data displayed on a log scale are based on log transformed regression coefficients.

A correlation analysis of the empirically derived MP, fractionated by the afore described physical properties and distinct sediment grain size fractions revealed strong correlations for HD particulate polymers (Fig. 4A,B). Station S8 and S9 were excluded as MP numbers were too low to calculate statistically robust percentage values of the respective MP fractions. HD particulate MP of sizes between 1000 and 5000 *μ*m correlated with the fraction of fine sand (125–250 *μ*m) with high significance (r = 0.96, p < 0.001, Fig. 4A). Likewise, HD particulate MP of 500 to 1000 *μ*m significantly correlated (r = 0.94, p = 0.002, Fig. 4B) with a grain size range between medium silt to very fine sand (16–125 *μ*m). The performed correlation analysis was based on grain size fractions classified by Udden and Wentworth^28^ and those that yielded most significant fits are presented. LD particulate polymers and fibres showed no clear correlation with either of these fractions. As mentioned before, the spatial distribution of LD polymers and HD fibres of the studied size range equalled each other. The best fit of this MP category was found with silt, although not significant (4–63 *μ*m, r = 0.51, p = 0.2, Fig. 4C). Instead, a significant correlation was found between LD particles in combination with all fibres and the fine sediment fraction of <63 *μ*m (r = 0.88, p = 0.002, dashed line in Fig. 4E).

The median grain size (d50) highly correlated with TMP abundance (r = − 0.9, p < 0.001); the finer the sediment, the more abundant TMP (Fig. 4D). The correlation was determined by the preponderance of HD particulate polymers (r = − 0.9, p < 0.001, Fig. 4D, grey diamonds). The sedimentary fine-grained fraction (<63 *μ*m) correlated with TMP (r = 0.86, p = 0.001, Fig. 4E), as also demonstrated by the high predictive power in Eqs. (1)–(3) of the GLM. No correlation was found between organic matter (total organic carbon, TOC) and TMP (SI, Fig. S4).

#### Theoretical validation

The transport behaviour of MP and non-cohesive sediment particles is controlled by their respective densities, shapes and grain sizes. Assuming a flat seafloor and spherical particles, the threshold bed shear stress *τ*_*cr*_ required to initiate movement on a sediment or MP particle can be calculated^11^:

4 $${\tau }_{cr}={\Theta }_{cr}g({\rho }_{s/p}-\rho )d$$

Here, *τ*_*cr*_ is the bed shear stress [Nm^−2^] exerted on the bottom at the initiation of movement that depends on the current velocity profile, water depth, water density and particle grain size, *g* is the acceleration due to gravity [m s^−2^], *ρ* is the density of water [kg m^−3^], *ρ*_*s*/*p*_ is the density of quartz (2.65 g cm^−3^) and plastic (assumed with 1.2 and 1.6 g cm^−3^), respectively. *d* is the grain diameter [m]. Θ_*cr*_ is the threshold Shields parameter, describing the equilibrium of force exerted on a particle by water movement and the counteracting force due to the particle weight. It is estimated from the Shields curve following Soulsby^11^. The density ratio *ρ*_*s*_/*ρ* may be directly important in controlling the threshold of motion, invalidating the above approximation of Θ_*cr*_ that was developed for density values of quartz grains. Such effects were measured for the case of large density differences (e.g. for different atmospheric pressures). However, for transport in water no impact of changing density ratios between 1.2 and 3 g cm^−3^ to the threshold of motion initiation have been reported^29^.

**Figure 5:**
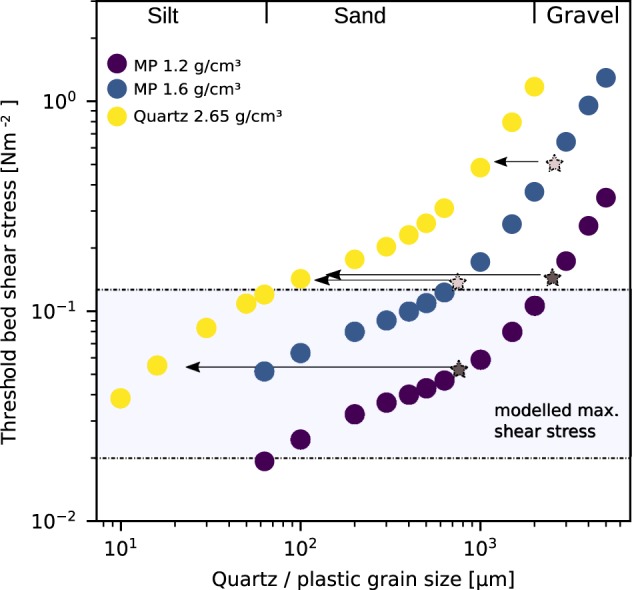
Threshold bed shear stress of quartz and plastic, HD ordinary polymers (average density 1.2 g cm^−3^) and paint resins (average density of 1.6 g cm^−3^) respectively. Stars and arrows indicate the equivalent quartz sediment grain and MP size (diameter) that is mobilised when subjected to the same bed shear stress. Stars are placed at the mean MP size fraction, 2500 and 750 *μm*, derived from the empiric analysis (Fig. 4A,B). The area between the dashed lines indicates the simulated maximum shear stress range (S2–S10) in the Warnow area based on a one-year average (see Hydrodynamic Model under the Materials and Methods section).

The results of the threshold bed shear stress calculation for particles of different grain size and density are shown in Fig. 5. The results show a non-linear relationship for the initiation of motion for quartz and MP particles of the same diameter. For large MP particles (2500 *μ*m as an average) of a high density (1.6 g cm^−3^), an initiation of motion comparable to quartz grains of 1000 *μ*m can be expected, while large HD MP particles of a lower density (1.2 g cm^−3^) would initiate transport with the fine sand fraction. Smaller MP particles (750 *μ*m as an average) of higher density initiate movement with quartz grains of the very fine sand fraction, while those of lower density mobilise with the medium silt fraction. Generally, the size difference between quartz and MP particles, mobilised under the same bed shear stress, decreases with increasing grain size and decreasing density difference.

The theoretical approximations are in general agreement with the empirical results, within the available accuracy limits discussed below. The analysed HD MP and sediment fractions show a shift in grain size by approximately one to two orders of magnitude. While effects of shape on particle movement were sparsely considered and a better distinction of MP grain sizes is required, the agreement of theoretical and available field data indicates that the observed particle size difference is the fundamental feature of HD MP dynamic. A better validation of the MP sediment relationship would require a higher resolution of MP size intervals. However, much higher MP abundances per sample are then required. Simulated shear stress data (see Hydrodynamic model) was retrieved to validate whether thresholds are sufficient to initiate motion of the particles measured. The transport of MP during average environmental conditions at the Warnow stations is limited (0.003 N m^−2^). The maximum bed shear stresses for a one-year modelling run (2014) were found to range between 0.02–0.11 N m^−2^ between stations, indicating that less dense or smaller HD particles are mobilised. Thus the bulk of MP transport in the Warnow estuary is likely to occur during extreme events causing increased bed shear stress. For LD MP, a comparison with established sediment dynamic models is not possible. While a correlation between LD polymers and sediment coarser than medium silt may have been not found due to the low number of LD particles in the sample distribution, no correlation is expected. LD polymers would initially stay afloat and thus have fundamentally different transport mechanics compared to sediment particles. LD may settle when influenced by external factors such as attach-detach cycles of biota^30^. This points towards a hydrodynamic relationship with fine-grained sedimentary components of a higher cohesive capacity i.e. sedimentation as a result of aggregation or flocculation, indicated by a significant correlation between LD and the fine sediment fraction (r = 0.77, p = 0.02, when all fibres included: r = 0.88, p = 0.002). Such a transport mechanism is supported by previous studies that found a TMP-TOC relationship^14,31^ for data sets dominated by sizes below the minimum size class analysed in the present study. This indicates that a correlation exists between TOC and TMP <500 *μm*.

Summarising, a fractionated granulometric normalisation based on mathematical correlations between the abundance of specific MP categories (defined by size, density and shape) and the reference sediment grain size fraction to eliminate hydrodynamic variability is the more accurate approach to account for different MP compositions within data sets. The application of single hydrodynamic parameters (e.g. d50, <63 *μ*m) for a granulometric normalisation is recommended as an approximation of MP abundances in case no further fractionation is possible. HD particulate MP > 500 *μ*m were best explained by the d50, based on the underlying direct size fractionated correlations (Fig. 4A,B). LD and fibrous MP (and supposedly TMP < 500 *μ*m) were better explained by the <63 *μ*m fine fraction. On the one hand, the application of LD MP and thus TMP abundances for the d50 normalisation within the studied size limits is questionable due to the absence of a clear correlation with a sediment grain size fraction (Fig. 4C) and median sediment grain size (r = − 0.64, p = 0.06, when all fibres included: r = −0.79, p = 0.01). On the other hand, the shown correlation between the <63 *μ*m fraction and TMP (or HD particles only: r = 0.86, p = 0.002) is likely to be based on a cross-correlation between <63 *μ*m and d50. A single accurate proxy is thus difficult to obtain for the entire MP pool (~10 *μ*m-5 mm, 0.89–1.6 g cm^−3^) and requires a selection tailored to the composition of the MP data set under investigation.

#### Residual analysis and MP re-assessment

TMP abundances as a function of sediment composition, such as median grain size, could explain a large degree of variability within the data set (decrease of mean squared error from 3.7 to 0.8, TMP log transformed). A residual analysis of the grain sized normalised TMP abundance values and the determined regression (base)line showed an independent distribution without any clear outliers (below ±3), although station S5 is close to this threshold (Fig. 4D, insert). Generally, such residual analysis can reveal stations that currently function as a sink or source (positioned below or above the regression line) to the investigated system at the time of measurement. In theory, granulometric normalised MP abundances would approach an optimum of zero residual deviance in a hydrological system of sufficient connectivity and steady state. Conclusively, granulometric normalisation of MP data is of importance when assessing MP abundances because site-specific differences were largely explained by grain size differences. Lacking this, variations might be misinterpreted as source load differences and hot spot areas. Concerning the fractionated granulometric normalisation approach it was observed that at stations with a low grain size, such as in S1 and S10, the exerted shear stress might be too low to transport certain size specific MP that entered locally. This could be the reason why these stations appeared as outliers in the size fractionated correlation analysis (Fig. 4A,B, respectively). This phenomenon of very fine grained areas hence low shear stress levels could represent a potential limitation in the MP projection based on shear stress correlates and needs further investigation, e.g. concerning the influence of sinking velocity.

### Implementation

#### Study site cross - comparison reveals essential parameters

The data of other study sites confirms the general relationship between MP and sediments as compiled and presented in Fig. 4E^9,14,16^. However, a comparison of MP contamination levels between different study sites is difficult due to a) different sampling and analysis techniques and b) often insufficient consideration of the hydrodynamic environment.

The MP - sediment relationship depends on the respective MP input level and spatio-temporal connectivity of the available sampling stations. A larger sample area coverage can cause a less prominent correlation such as in Strand *et al*. and Ballent *et al*.^9,14^. Similarly, less significant fits can occur when temporal connectivity is not fully guaranteed. Highest correlations were consequently achieved by Vianello *et al*.^16^ and the present study, both based on small scale well-connected sampling areas and periods. Deviations in the course of the shown graphs (4E) can occur due to different analytical preparation steps applied. A table comparing the displayed studies in different systemic parameters is provided in the SI, Table S1. Varying MP size thresholds as well as different density separation thresholds were applied during MP isolation by these studies. If the mentioned systemic differences were resolved an accurate comparison of the (normalised) MP contamination level between sites could be made. An additional common constraint of studies that reported the absence of a relationship between MP and sediments, is a missing coverage of large variability ranges of sediment grain sizes^17,18^ or complete exclusion of the fine fraction^17^, partly owed to a low sample sizes. A sample coverage spanning a sufficient range of grain sizes, representative for the region of interest, is thus essential to determine the MP contamination level in sediments. The sedimentary environment in which MP resided in the Warnow estuary spanned a reasonable diversity of sediment types, from clayey silt and very silty sandy mud to slightly silty sand (classified according to Flemming^32^) with a d50 range across 100 *μ*m. Some of the above referenced studies did not verify MP suspects via spectroscopic or other chemical test measures (SI, Table S1) which allows no unambiguous and thus sound identification and quantification, especially in the small size range^33^.

#### MP contamination level comparison to Baltic Sea basins

Two Baltic Sea basin sediment samples were analysed and grain-size normalised to be compared to the MP contamination levels found in the Warnow estuary. These samples, together with sediment trap samples, revealed valuable information on the connectivity of the two systems in terms of MP bed load transport and major transport pathways to this environment.

**Figure 6:**
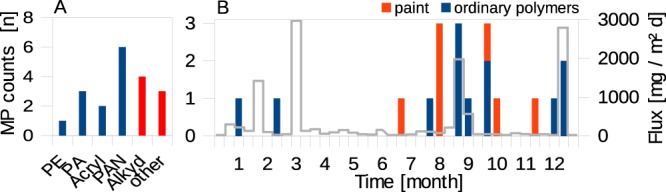
Ordinary polymer (blue) and paint resin (red) distribution found in the Arkona basin sediment trap samples. (**A**) cumulative and **(B)** over time. **(B)** Numbers on the x-axis represent the time in months over the sampling year from December 2012 until January 2014. The grey line illustrates the sediment flux (<400 *μ*m). Ordinary polymers are all of a fibrous nature.

No plastics within the analysed size range were found in the sediment trap samples from the Gotland basin over the entire period of nearly 10 months (excluding 9 PET fibres counted as contamination suspects, see Text S1). In the Arkona basin samples, a cumulative number of 12 ordinary polymers, all of which were fibrous, and 7 paint resins were identified (Fig. 6). Assuming a constant intra-annual MP sedimentation rate, this amounts to 0.1 m^−2^ d^−1^, which sums up to 37 m^−2^ per year (Fig. 1B). The maximum entry within a sampling interval of 10 days amounted to 3 MP. There were slightly more MP during the late summer and autumn months which generally coincides with a higher sedimentation rate of natural particles (Fig. 6B). However, differences between time intervals were marginal due to the small sample size and could well derive from increased local MP input, as, for instance, from maritime activities. The observation is made that paint resins in contrast to ordinary polymers descend independent from the natural particle flux, indicating a sea-based origin independent from natural particle transport routes. Sedimentation rates were based on the <400 *μ*m fraction, which is used as a proxy for the total material flux.

The polymer composition in the Arkona basin sediment trap sample showed that only approximately 5% of MP (i.e. one PE fibre) belonged to the group of LD polymers (Fig. 6A); a percentage comparable to the Warnow estuary. One paint resin and one PA fibre could be identified in the Arkona basin sediments, being equivalent to 5 kg^−1^ DW (Fig. 1B) and largely matche the polymer composition found in the water column. Also MP abundances between the two interlinked compartments show reasonable consistency as discussed in a hypothetical comparison as follows. Reported mean sedimentation rates in the Arkona Basin of 3 mm m^−2^ y^−1^ ^34^ imply that the taken sediment sample comprises layers equivalent to the last 33 years. Based on the MP sedimentation rate from sediment trap sampling, 1233 MP m^−2^ could have sedimented during this time period based on current MP sedimentation rates. An extrapolation of the MP abundances found in the Arkona sediment sample of 5 kg^−1^ DW equal 222 MP m^−2^. Based on knowledge from sediment records, it seems reasonable to assume that current MP sedimentation rates rose with production volumes during the last decades^35^. Then, the cumulative number of sedimented MP would be about half of that currently measured and, thus, lied within the same order of magnitude.

The calculation of MP sedimentation rates in the sea basins paired with the time integrated abundances found in the sediment below indicate a direct link between water column borne and benthic MP in this region. This would imply that a majority of the local MP burial originated from vertical deposition of particles rather than horizontal near-bottom transport, suggesting a sea-based origin or long distance transport of particles in suspension. The comparison of the MP abundances found in the Baltic Sea basins normalised to grain size (Fig. 4D,E, crosses) showed a contamination level of one to two orders of magnitude below that of the estuary. Under the restriction of the low sample size, these comparative data points shall exemplify the impact of the granulometric normalisation on the interpretation of MP contamination levels (methods identical). Otherwise, it would have been within the same range of that of the estuary. A larger data set (across a larger grain size range) is nevertheless needed to draw a grain size corrected MP contamination curve and thus determine the contamination level of the Baltic Sea region.

*MP sinks*: Sorting of particles in suspension along their deposition velocities is ultimately influenced by the environment’s turbulence level. With a diminishing level of turbulence along a depth gradient from coastal environments, the deep sea basins are expectantly a sink for very fine sediments (<63 *μ*m) and thus MP that correlate with that fraction (fibres, LD polymers and MP < 500 *μ*m). This is in coherence with our findings of a predominance of fibres and generally very small numbers of MP > 500 *μ*m in the basins. The absence of HD ordinary particles, suggests no significant long distance transport to the sea basins but their sedimentation closer to the source. Paint resins with a higher relative threshold bed shear stress and sinking velocity as well as their sedimentation decoupled from natural particle flux suggests a sea-based entry. That fibres can reach the Baltic Sea basins from land-based sources was recently demonstrated in a modelling study^7^. Our finding, that fibres dominated sea basin sediments, echo the MP composition found by another study^3^, (paint resins excluded). The function of low energetic environments as a sink for smaller MP (<500 *μ*m) and its scale should be focused on in future studies. Particles of sizes where density-determined advection becomes negligible compared to turbulence-driven diffusion are considered equally distributed throughout the sea^26^. Then other mechanisms gain influence: conglomeration of suspended material rises the particles deposition velocity and consolidation and cohesion of deposited material increases the threshold shear stress necessary to initiate motion. Once deposited, (Baltic) sea basins foster an ultimate burial of MP, due to the relatively low resuspension probability. In the Baltic, only rare inflow events of dense, saline North Sea water or strong cyclonic winds could temporarily resuspend MP^36^.

### Backtracking point sources

A large-scale correlation between population density and MP along river shores was recently demonstrated^37^. In the present study, sediment samples were taken in a relatively dense spatial resolution, which allowed to reveal direct sink source relationships. As a result, major point sources were backtracked to the WWTP and harbour areas.

Despite the generally high filtering efficiency of MP by WWTPs, absolute release numbers were found to be significant^38^. The relatively high threshold shear stress required to transport paint resins explains their most significant source backtracking curve within the data set (Fig. 3A). The occurrence of paint resins coincides with previously documented elevated heavy metal burdens of copper, cadmium and lead in the estuary^39^. Cross-correlations with other emitters of heavy metals are possible. However, heavy metal leaching from the paint matrix and their function as transport vehicle of contaminants to the environment is unquestioned^40^. Copper, for instance, is an active ingredient in most antifoulant paints and a chromophore complex constituent of many blue and green pigments^41^, reaching highest concentrations compared to other heavy metals.

The found micro PS beads are likely to be emitted by nearby industries, as a contribution of this sector has been shown to exist^9,42^. In the present study, parameterisation and thus allocation to the industrial sector were, however, complicated due to the broad application possibilities of these products. PS ion exchangers are being used for water purification, desalinisation, softening, demineralisation and micro-fouling control and are used predominantly in metal or pharmaceutical sectors. It is especially due to the above mentioned industrial sectors that billions of m^3^ of untreated waste water enter the aquatic environment annually in Germany^43^. In an Austrian case study^44^, the generous thresholds that are set for industrial MP discharges were criticised. Similar ‘spherules’ in water samples were identified along the Rhine river which occurred at very high frequencies (overall average of 60%), especially in areas of high industrial density^42^. Proportions (58% overall), morphology (opaque, 300–1000 *μ*m in size), their polymeric assignment to cross-linked PS and the proximity to industrial areas compare with our findings in Warnow estuarine sediments. Their apparent presence in both compartments, the sediments and the water surface, might be explained by the inclusion of gas bubbles within the spherules as occasionally observed by the mentioned study^42^. Ageing effects, described for ion exchangers as the oxidative breakage of cross-linkers past water uptake, can also cause swelling and softening^45^. The general dominance of the PS based ion exchanger beads shows that they constitute a so far largely ignored MP species of potentially very large quantities in urban waters.

## Conclusions and Prospects of Granulometric Normalisation

Particles, whether they are of clastic sediment or synthetic polymeric nature are principally governed by the same environmental laws. The presented direct correlations in transport - deposition behaviour between the two materials can be used as a foundation for an improved estimation and projection of the MP distribution in sediments confirming our initial hypothesis. Fractionated granulometric normalisation based on mathematical correlations between specific MP categories and the reference sediment grain size fraction to eliminate hydrodynamic variability is highly recommended. This has implications on the interpretation of former study results, concluding that without correction for variability of natural particle composition in consequence of hydrodynamic differences, rational comparison of MP abundances between samples is impeded. We propose this as a basis for the prediction of MP distribution from small precursory MP deposition data sets by using spatial sediment grain size data along with data on local sources. Considerations on spatio-temporal connectivity of the system studied and heterogeneity of the sedimentary matrix are pivotal for a sound and comprehensive assessment. Resolving the physical fate of MP in the aquatic environment can be approached by building upon the extensive knowledge of sediment transport mechanisms. Using sediment grain size as a proxy, identification of pathways, sinks and sources of MP on a regional and potentially global scale are achievable objectives. Changing anthropogenic influences, over space and time, can subsequently be determined, which is of direct importance when applied as a stratigraphic indicator of the anthropocene^46^. In a nutshell, a close look at sediment distribution maps is of importance prior to sampling as well as during data interpretation when assessing an area’s MP contamination level.

The developed MP-sediment relationship should be understood as a first approximation derived from field data. The basic calculations of sediment and MP dynamics and the comparison to normalised data of previously published data supports the idea of a general applicability of the fractionated granulometric normalisation. However, further validation and extension by larger data sets covering larger MP size ranges, analysing the influence of shape in more detail and testing the relationships found in areas with similar as well as deviating hydrodynamic conditions, such as fluvial and marine, are needed. In areas where other mechanisms determine particle sorting (i.e. sinking velocity), correlations might deviate. It is questionable whether beaches and shorelines reflect the same direct linkage between MP and sediment grain size^8,47^. The more complex distribution behaviour of LD polymers should be a future research focus to complete our understanding of MP transport and deposition patterns.

## Materials and Methods

### Sample collection

#### Warnow estuary

Sediment sampling in the Warnow estuary followed a transect, from past the sluice gate (S1) in the centre of the city Rostock, downstream to approximately 2 km past the estuary where it disembogues into the Baltic Sea (S8, S9). A total of 9 stations were sampled along the lower Warnow, plus one station (S10) in the adjacent side arm Alter Strom connected via the Baltic Sea. In close intervals of 0.8 to 5 km, sampling was conducted from aboard a boat by deploying a Van-Veen grab (which covers an area of 0.04 m^2^ and has a maximum capacity of 5 l). Wet weights ranged from 1550 to 2700 g. At two stations a total of three replicates were taken and analysed exemplary to evaluate local variability. The sampled sediment was then transferred into a 1.5 l glass jar with the use of a metal bowl and spoon, which were thoroughly rinsed with micro-filtrated water (MilliQ) beforehand. The samples were stored in a dark room at a temperature of 4 °*C* until processing.

#### Arkona and Gotland basin

Sediment trap sampling: Sediment trap sampling was part of a larger bentho-pelagic monitoring program and comprised two series, one in the Arkona, the other in the Gotland basin, with individual sampling intervals (1–20) of 10 and 14 days, respectively (location information: SI, Table S2). The round and funnel-shaped inlet of the sediment traps spans 80 cm in diameter, covering an area of 0.503 m^2^. The sediment trap content was collected in a 400 ml PE vessels and subsequently sieved, as only the fraction >500 *μ*m was investigated for this study. The remains were fixed in formaldehyde and kept in 20 ml glass vessels.

Sediment sampling: Sediment samples at the two Baltic Sea stations (same locations as sediment traps) were taken with a box corer during the Poseidon cruise 488 in 2015. The box corer covers an area of 15 × 15 cm and a sampling depth of 10 cm was attempted. 1 kg wet weight from each location, comprising 500 g of two separate samples, taken in parallel, was the basis for later analysis.

### Sample preparation

#### Sediment samples

DW analysis was conducted by determining the weight differences of a defined amount of homogenised wet sediment subsample between pre- and post-drying in a drying closet, at 60 °*C* for 48 h, until no further weight reduction was measurable. Wet sediment samples were density separated using a MicroPlastic Sediment Separator (MPSS, Hydro-Bios). The instrument is entirely made of stainless steel and glass, except for EPDM o-rings and PTFE hoses and sealings. The MPSS has a documented recovery rate of 100% in the analysed size class. A detailed description of the MPSS^48^ and appropriate protocols^49^ are published.

In this study we used sodium polytungstate (TC-Tungsten Compounds) at a density of approximately 1.8 g cm^−3^ as a density separation solution. A methodological remark on the recommended density separation threshold that includes paint resins is available in the SI, Text S3. Each sediment sample was added to 4l of prefiltered sodium polytungstate in the MPSS with a metal spoon. By means of a vacuum pump, the density solution was transferred from a stainless steel barrel to the sediment container, passing two prefilters of 10 and 5 *μ*m. A rotor (20 rpm) at the bottom of the container stirs the sample to free potential MP and pass them into the conical stand pipe and then into the dividing chamber for final separation. Larger floating pieces (>1 cm) such as shells or pieces of wood were removed (rinsing ensured no MP loss) as they can cause clogging of the dividing chamber. In order to avoid precipitation reactions, causing the clogging of filters and leakage, the pH had to be kept at an optimum of 3 to 3.4. This was achieved by adding prefiltered 37% hydrochloric acid after each passage when the solution had been in contact with the sediment at higher pH. Density was checked regularly and readjusted if necessary. The separation process lasted 24 h while the MPSS hull was occasionally knocked, to limit the possibility of MP adhering to the walls. Following this, the separation chamber was closed and dismantled and the supernatant transferred into a beaker glass. The remaining residues in the separation chamber were rinsed with MilliQ into a second glass beaker. After each sample run, the sodium polytungstate was filtered through a 15 *μ*m mesh back into the storage barrel. The liquids were collected to be recycled by the producer. Prior to each new sample treatment, a cleaning sequence guaranteed a contamination free MPSS. For general contamination prevention measures and control testing, see SI, Text S2.

Since this study intended to examine the larger fraction of MP, the supernatant was passed through a stainless steel sieve with apertures of 500 *μ*m. Samples not directly analysed, were stored in beakers filled with MilliQ and covered with aluminum foil. The sample was subsequently pre-analysed under the microscope (Zeiss Stemi 200, magnification: 6.5 to 50×) in a custom-built Bogorov chamber (20 × 20 cm). MP suspects were manually isolated according to defined visual criteria^50,51^. These suspects were then placed between two glass slides, which were then kept bound together with parafilm, pending spectroscopic analysis. The sieve was thoroughly scanned for potential MP remains.

After density separation, some of the samples (S1, S2, S3, S8, S9) underwent further treatment in order to reduce organic material content and ease subsequent visual and spectral analysis. An array of treatments with SDS, enzymes, sodium hydroxide (NaOH), hydrogen chloride (HCl) or a repeated density separation were applied depending on the degree of biogenic or silicate debris^52^. All applied chemicals are known to be non-destructive to MP^53^.

#### Sediment trap samples

Some of the Gotland samples (1–2, 3–4, 7–9, 10–12, 13–15, 16–17) were pooled because of their low sample volume. In total, 49 subsamples were taken. Sediment trap samples were transferred to glass petri dishes and examined for MP by means of a binocular microscope (Zeiss Stemi 2000, 6.5–8x magnification, 50x for a detailed study of the particle morphology) from top to bottom in horizontal lines. This was then repeated up to three times in different orientations of the petri dish. In case of large volumes, the sample was subdivided. By means of fine tweezers with 0.05 × 0.01 mm tips, isolated MP were transferred in 1.5 ml PE Eppendorf-tubes previously filled with 700 *μ*l sample water. As the removal of biofilms and other material is generally recommended to ensure best possible spectra quality during FTIR and Raman spectroscopy^33,54^, the isolated MP suspects underwent a 72 h purification treatment by adding 750 *μ*l of 30% hydrogen peroxide (H_2_O_2_) solution. Both efficiency and resistance by plastic of the oxidising agent is known from other studies^55^. Temperature rise of the exothermic reaction was ensured to be minimal^56^. Via bottle top filtration, the samples were transferred onto PC filters of 3 *μ*m pore size (47 mm in diameter) which were then separated using filter paper stored in petri dishes until further analysis. MP suspects were photo-documented in a second binocular run (Zeiss Discovery V.8 Stereo with camera module AxioCam ICc 3, Software Axio Vision, 16–128x magnification), and pregrouped into plastic or unknowns. Subsequent spectroscopic analysis would correct for false positive misidentifications.

### Chemical characterisation via *μ*-ATR-FTIR and Raman-spectroscopy

All MP suspects were analysed via micro-attenuated total reflection - Fourier transform infrared spectroscopy (*μ*-ATR-FTIR) as single point measurements. A Bruker Vertex 70 FTIR- spectrometer coupled with a Bruker Hyperion 2000 FTIR-microscope equipped with a 20x ATR objective with a germanium crystal and a Mercury Cadmium Telluride single element detector was used. Measurements were taken with a resolution of 4 cm^−1^ in a wavenumber range of 4000–600 cm^−1^ with 100 scans per sample. The contact area of the germanium crystal approximates 25 × 25 *μ*m. The FTIR detector requires liquid nitrogen for cooling and this must be filled 30 minutes before operation and repeated every 5–6 hours. Background spectra were regularly measured against air with the same setting as described above. Appropriated signal-to-noise ratios required a close contact between the ATR crystal tip and MP suspect situated on the glass slide. Between measurements the ATR crystal was cleaned with ethanol to avoid cross-contamination.

The software Opus 7.5 (Bruker Optics) was used for spectral measurement, processing and evaluation. Multiple reference libraries were indexed: ATR-FTIR Library complete Volume 1–3, Hummel Industrial Polymers Vol. 1–3, Bruker Optics, as well as internal IPF libraries. Plastic-positive spectra as well as some exemplary non-plastic spectra were archived and are available upon request. For items yielding insufficient spectral quality (about 5–10% of the samples) complementary measurements were either taken via Raman-microspectroscopy or were excluded. Single point measurements by Raman-microspectroscopy were performed as described in Käppler *et al*.^54^.

### Morphological characterisation

Potential MP items were categorised according to shape, colour and size. MP length were digitally measured via GIMP (version 2.8.16) across their longest dimension and categorised into 500–1000–2000–5000, >5000 *μ*m intervals. In case of repeatedly occurring identical items, a subset was measured spectroscopically.

### Geological and chemical analysis

Grain size distribution was analysed in two sample replicates with the laser-sizer CILAS 1180 with ultrasound applied. For station S5–S8, grain size data is based on only one measurement due to problems during sample splitting, which could not guarantee grain size independent separation. Prior to analysis, the samples were homogenised. A 30% H_2_O_2_ pretreatment ensured organic matter-free material. Organic and inorganic carbon, nitrogen and CaCO_3_ determination was conducted on lyophilised sample material using elemental analysers multi-EA 2000 (Analytik Jena) and EA 1110 CHN (CE-instruments). These granulometrical methods are well established in geological studies^57^.

### Hydrodynamic model

For data retrieval of simulated shear stress values from the Warnow estuary model simulations were carried out with the help of the three-dimensional coastal ocean model GETM (General Estuarine Transport Model^58^). It calculates time series of salinity, temperature and current velocity among others, on a high-resolution grid with 20 m horizontal resolution. As GETM has been successfully applied in numerous studies (e.g. in^59^), particularly in the Warnow estuary^23,60,61^, only very fundamental information on the model is provided. For more detail the reader is referred to the previously mentioned publications. Here, the same underlying model configuration has been used with a state-of-the-art turbulence closure scheme GOTM (General Ocean Turbulence Model) and a second-order advection scheme (SUPERBEE) to reduce numerical diffusion and fully conserve mass, energy and momentum. Meteorological forcing is calculated from output of the German Weather Service Local Model (DWD-LM) with a horizontal resolution of 7 km. Sea level, temperature and salinity at the open boundary are generated by larger scale outer models of the western Baltic Sea (see^59^ for details) and time series of Warnow river discharge are provided by the Federal Maritime and Hydrography agency. The barotropic processes are captured in terms of sea level with a root mean square error of 0.08 m and bottom temperature is reproduced with a r.m.s.e. of 0.82 °C.

### Statistical analysis

Regional abundances are presented as median value with the 25 and 75-percentiles [0.25–0.75] due to non-normalility. Replicated samples are presented as mean ± standard deviation (SD). Two-tailed Pearson correlation coefficient *r* was used to test for the degree of association in case of linear relationships; otherwise Spearman’s rank *r*_*s*_ is mentioned when applied. In figures containing regression analysis the coefficient of determination *r*^2^ is given as a measure of Goodness-of-fit (least square) together with the fitted equation. Outliers were based on studentized residuals with the threshold at ±3.

As the MP abundance data *μ*_*i*_ [kg^−1^ DW] analysed with the GLMs was both not normally distributed and integer based, the poisson link function was chosen:

5 $$log(\hat{{\mu }_{i}})={\beta }_{0}+{\beta }_{1}{x}_{i,1}+{\beta }_{2}{x}_{i,2}+\ldots +{\beta }_{k}{x}_{i,k},i=1,..,10$$

After a first preselection, a set of *k* predictor variables *x*_*i*_ were tested. Unstandardised *β* coefficients were calculated to estimate parameter influence. Both backward and forward simplification approaches were applied to achieve best model fit. Whether to include or remove a term was evaluated by means of the AIC, deviance and the significance of the standardised regression coefficients (p < 0.05). Overfitting was addressed by using the adjusted *r*^2^. Exploratory data analysis (EDA) assisted the decision process to, for instance, avoid colinearity among variables which would reduce statistical power. Colinearity was resolved either by using a combination of the two variables for logical relationships or exclusion of one of them. Model validation was conducted via residual analysis, visualised in the probability plot of the Pearson residuals for standard normal distribution and the plot of residuals vs. fitted values. The GLM was built in matlab (version 2017b).

### Geospatial analysis

Source related predictor variables mentioned above were generated using the nearest neighbour analysis and intersection geoprocessing tool in QGIS^62^ using the UTM-33N (EPSG:7417) projection. Data from potential diffuse sources were determined within a buffer zone of 1000 m (radius) which was a trade-off between the catchment zone and practicality, as this avoided overlapping of influence zones. Point sources were measured as distances. Diffuse sources were counted within a 1000 m radius. Parameterisation was based on a qualitative assessment of the present estuary with regards to potential MP emitting sources, as they are generally widely discussed in the literature^63^. This was assisted by EDA. It is noted that the data set may not be complete and data retrieval is dependent on both the type of reference and the method of choice. A transfer of the model to other locations would possibly require incorporation of additional parameters of relevance and/or to dispense with others.

## Supplementary information

Supporting Information (SI)
