# Author Correction: Tracing microplastics in aquatic environments based on sediment analogies

**DOI:** 10.1038/s41598-019-54997-z

**Published:** 2019-12-06

**Authors:** Kristina Enders, Andrea Käppler, Oliver Biniasch, Peter Feldens, Nicole Stollberg, Xaver Lange, Dieter Fischer, Klaus-Jochen Eichhorn, Falk Pollehne, Sonja Oberbeckmann, Matthias Labrenz

**Affiliations:** 1 Leibniz Institute for Baltic Sea Research Warnemünde (IOW), Seestraße 15, 18119 Rostock, DE Germany; 2 Leibniz Institute for Polymer Research Dresden (IPF), Hohe Str. 6, 01069 Dresden, DE Germany

Correction to: *Scientific Reports* 10.1038/s41598-019-50508-2, published online 23 October 2019

The original version of this Article contained typographical errors.

In the Abstract

“In Warnow estuarine sediments (Germany) we found significant correlations between high-density polymer size fractions (≥500 mm) and sediment grain size.”

now reads:

“In Warnow estuarine sediments (Germany) we found significant correlations between high-density polymer size fractions (≥500 µm) and sediment grain size.”

In the Results and Discussion section under the sub-heading “MP abundance and composition in the Warnow estuary”:

“The Baltic Sea opening had comparatively low abundances of 2[2-2] kg^−1^ DW (S8, S9), whereas the Alter Strom (adjacent side arm of the Warnow estuary) revealed highest levels of 379 ± 28 g^−1^ DW (n = 3, S10) (Fig. 1A) of the area studied.”

now reads:

“The Baltic Sea opening had comparatively low abundances of 2[2-2] kg^−1^ DW (S8, S9), whereas the Alter Strom (adjacent side arm of the Warnow estuary) revealed highest levels of 379 ± 28 kg^−1^ DW (n = 3, S10) (Fig. 1A) of the area studied.”

In the Results and Discussion section under the sub-heading “Fractionation of MP species based on physical properties”:

“In the Alter Strom, fibres reached abundances of 84 ± 45 g^−1^ DW, roughly equalling particulate ordinary polymers.”

now reads:

“In the Alter Strom, fibres reached abundances of 84 ± 45 kg^−1^ DW, roughly equalling particulate ordinary polymers.”

In the legend for Figure 1

“Representative replicates (n = 3) were available for station S2 and S10 amounting to 78 ± 10 g^−1^ DW and 379 ± 28 g^−1^ DW, respectively.”

now reads

“Representative replicates (n = 3) were available for station S2 and S10 amounting to 78 ± 10 kg^−1^ DW and 379 ± 28 kg^−1^ DW, respectively.”

In the legend for Figure 2

“The black line indicates the transition from LD to HD polymers determined by the density of water (~1 g m^−3^).”

now reads

“The black line indicates the transition from LD to HD polymers determined by the density of water (~1 g cm^−3^).”

These errors have now been corrected in the PDF and HTML versions of the Article.

