# Supplementary material for: Tracing microplastics in aquatic environments based on sediment analogies
Source: Sci Rep. 2019 Oct 23;9:15207. doi: 10.1038/s41598-019-50508-2 (PMC6811616; doi:10.1038/s41598-019-50508-2)
Supplement: Supplementary file 1 — Supporting Information (SI) [file 41598_2019_50508_MOESM1_ESM.pdf]

Supporting Information (SI)

Tracing microplastics in aquatic environments based on sediment analogies

Kristina Enders, Andrea Käppler, Oliver Biniasch, Peter Feldens, Nicole Stollberg, Xaver Lange, Dieter Fischer, Klaus-Jochen Eichhorn, Falk Pollehne, Sonja Oberbeckmann and Matthias Labrenz

Files in this SI Appendix: Text S1 to S3, Figures S1 to S4, Tables S1 to S3

Text S1. Composition and distribution of MP categories

Paint resins. Paint resin distribution was subject to great fluctuations and local hotspots appear with highest abundances in the Alter Strom of 210 ± 69 kg^{-1} DW. The background level of paint outside the hotspot areas can be described as relatively constant and ranges between 0 to 10 kg^{-1} DW. Paint resins were grouped according to most frequently appearing categories. Alkyd resin (which incorporates acrylic resins as they are often applied in combination and also exhibit spectral similarities) comprising 90% of all measured paint resin particles, was by far the most dominating category. Polyurethane (PU) resin was a separate group with a percentage frequency of 2%. PU-alkyd combinations (urethanisied alkyds) were common and classified as alkyd. With 6%, epoxy resins were the second most common group. Slightly less than 2% could not be clearly assigned to any of the above. However, spectral bands showing indications of paint resin origin and the presence of typical morphological features were deemed sufficient criteria to include these particles as paint resins.

PS beads. PS beads appeared in a very patchy distibution pattern, only at station S1, S2 and S5 ranging from 24 ± 21 to 317 kg^{-1} DW. For some PS beads found at station S2, very high spectral quality with correlation scores between 80% and 97% enabled a precise allocation as either sodium polystyrene sulfonate (SPSS) or calcium polystyrene sulfonate (CPSS), both being ionomers. This revealed their identity as ion exchangers. For these PS beads, a colour gradient from transparent to orange to brownish to black and sizes approximating 500 to 900 μm were detected. Station S1 solely contained opaque non-transparent beads that matched with pure PS within a slightly broader size range of 500 to 1100 μm. A mixture of both types along with polystyrene divinylbenzene (PS-DVB) occurred very abundantly, with 317 kg^{-1} DW recorded at station S5 (Fig S1). Due to the high numbers of PS beads and the occurrence of spectral masking, only subsets were spectroscopically examined ($N_{spectra}$, see Fig. S1). Others were identified based on identical morphological features wherefore relative abundances of the PS bead species were not ascertainable. In some cases, clear identification was limited either due to the masking by foreign spectra of water or other inorganic residues. It is not unusual that ion exchangers have a high moisture retention[48], which might explain this spectral overlay as well as the different degrees of softness found. Depending on the manufacturer and age the crosslinkage and water load of the ion exchangers can vary vastly and as a result its density. Therefore an accurate specification of the density cannot be made. Since the basis polymer is PS, they were categorised accordingly. This assumption is acceptable as for the latter correlation analysis no differentiation between the different HD polymers was made.

Ordinary polymers. Ordinary polymers were most abundant at station S3, approximately 200 m downriver past the wastewater treatment plant (WWTP), amounting to 93 kg^{-1} DW. Also, the two stations S4 and S5 reached elevated numbers in both particles and fibres of 19 kg^{-1} DW and 9 to 10 kg^{-1} DW, respectively, compared to all other Warnow samples. In contrast to LD, HD ordinary polymer numbers dropped quickly after station S3. Apart from this elevated region, numbers of ordinary polymers remained relatively low between 4 to 9 kg^{-1} DW within the estuarine transect and only 1 kg^{-1} DW within the Baltic Sea opening past the estuary (S8, S9). Fibres found in the Arkona basin fibres by polymer composition showed indication of maritime sources: PA, the material most fishing ropes and nets are made of. Included in this group is the high tech fabric poly paraphenylene terephthalamide (aka Kevlar) which finds many applications in the marine sector due to its thermal and fire resistance and lightweight strength. Further, acrylates (acryl) for fishing ropes and PAN primarily found as copolymer also has various marine applications (sails, fishing rods).

Text S2. Contamination control

Results. Controls taken alongside the sediment trap samples only contained cellulose fibres (resulting from a heavy usage of paper towels in the laboratory) and no plastic polymers. Additionally, the sediment trap control bottle from the field contained no plastic in the analysed size fraction, giving an indication of a contaminant-free work flow. By contrast, the sediment sample control representing the MPSS passage revealed three blue fibres comprised of polyesters (PET). PET fibre abundances found in the Warnow sample set correlate significantly with overall fibre abundances ($r = 0.71$, $p = 0.02$) suggesting that contamination of these PET fibres is marginal, as an otherwise randomised or constant background value is expected. Moreover, the likelihood to find PET fibres among sediment-related samples is generally rather high, as it is a HD polymer (1.37 g cm^{-3}) and among the major polymer types produced[66]. Furthermore, their coinciding appearance with other fibres, especially at S5 with high PET numbers of 15 kg^{-1} DW, as well as highest values in the Alter Strom, indicate a rather low contamination level. The widespread occurrence of PET in the sample set, conversely, usually within the same colour regime (transparent- blue - black) can be interpreted as an indicator of contamination. As the limited number of controls do not allow for a quantitative assessment of

contamination, a conservative interpretation requires an entire exclusion of PET fibres from the final results. This includes the data from the sediment trap samples as the surrounding conditions (operators, laboratory facility) did not change and air-born sources are most probable. Although the purification process of the sediment trap samples included less intensive chemical treatment, PET appeared frequently across the samples sets, with more than half (Akrona basin, $n_{PET fibre}$ = 17) or all (Gotland basin, $n_{PET fibre}$ = 9) of the polymers identified being assigned to this polymer type.

A second polymer type was eliminated from the results retrospectively; PTFE. This is because the conclusion must be drawn that these MP originate from the treatment within the MPSS, as several parts, such as within the separation chamber and the outlet valve, are made from this polymer. We observed that, without exception, all PTFE particles found have the same appearance (transparent-whitish, within the same size class, morphologically similar). The low but non-correlated and yet widespread occurrence throughout the sediment samples (including Arkona basin sediment sample) and absence in the sediment trap samples which did not undergo the MPSS treatment strengthens an MPSS-associated PTFE contamination. The pH adjustment of the sodium polytungstate solution was done in a Schott flask with a PTFE screw cap which was subsequently refilled into the MPSS. Abrasion of PTFE particles is possible. Although, the sodium polytungstate solution has a lower density than average PTFE ($2.1 - 2.3$ g cm^{-3}), surface tension effects could lead to the flotation, and consequent retrieval, of these small particles[67].

Contamination prevention and controls - Methods. To comply with requirements of quality assurance, both the application of control samples and best practice contamination prevention measures throughout both lab and field work were ensured. A cellulosic laboratory coat and nitrile gloves were worn at all times. Prior to use, all instruments and vials were sanitised with tap water and rinsed with deionised and micro-filtrated water (MilliQ, 0.2 μm). Where possible prefiltered 70% ethanol was used for decreasing the water drops surface tension in which plastic particles or fibers potentially could remain. Sieves were additionally ultra sonificated (up to three times for 10 minutes). Frequently used metal and glassware were flamed. Filtration measures were undertaken within a laminar flow chamber in order to avoid air-born contamination. During microscopy, petri dishes were kept closed and the Bogorov chamber was covered with aluminum foil while awaiting further analysis.

The sodium polytungstate solution was recycled each time by filtration through a 15 μm gaze, ensuring that the density separation solution was free of contamination within the detection limit. Given the used separation density, flouropolymeric compounds such as polytetrafluorethylen (PTFE, 2.2 gcm^{-3}) is not accounted for as the density would need to be increased significantly. The application of PTFEs as coatings of containers, hoses and sealings, bears the potential for unknown systematic contamination, as was the case in the present study. There are multiple reasons for excluding PTFE from particle-based study designs. Production volumes are proportionally low and PTFE MP probably enters the environment preferentially in the lower micrometer or nanometer size range since it has become highly relevant as nano-coatings[68]. The omnipresence of perfluorochemicals (PFCs) in nature along with the ecotoxicological risk have been studied extensively. Specific mass-based detection methods of PFCs already exist[69].

In general the use of plastic items was avoided where possible. Restrictions are to be noted during sediment trap sampling, as the aperture is partly made out of plastic or coated with paint. The contamination risk for particles > 500 μm is, however, calculable as MP collection bottles closed underwater and were not opened until analysis. It is expected that any system-caused MP contamination would be identifiable by a correspondence between the characteristics of any used and found particles or a repetitive occurrence of the same kind of particles throughout the sample set.

Control steps accounting for contamination during the processing procedures were taken according to the same procedures as the sediment samples or sediment trap samples were processed. In parallel to the sediment samples (n_{Warnow} = 14; $n_{Arkona+Gotland}$ = 2), a total of three controls, where two were covering the MPSS treatment and another one the exposure time during the visual identification process in the Bogorov chamber, were acquired. The degree of contamination during the sediment trap sample processing ($n_{Arkona+Gotland}$ = 2 with 18 - 20 subsamples each) was evaluated by means of three controls in total. One separate collection bottle in the sediment trap set-up accounted for possible contamination during this part of the field sampling process. The bottle was filled with MilliQ water while sampling time and bottle lid opening and closing movements resembled the actual sampling. In this way, potential abrasion from the bottles entirely comprised of PE would be detected.

Text S3. Methodological remark on density separation threshold

The crux of MP field studies is the missing standardisation of MP isolation techniques. Density separation is most commonly used, however, setups greatly vary and the critical density is often below that of many HD polymers which prevents a detailed distribution analysis of the full spectrum of synthetic polymers[70]. The authors consider a density separation threshold of 1.8 g cm^{-3} as scientifically reasonable, as it includes the full spectrum of commonly excepted plastic polymers. It also accounts for the majority of dried paint resins[29], which were calculated to posses an average specific gravity of approximately 1.6 g cm^{-3}. The chemical composition of paint resins: polymer base resin, pigments and additives, resembles that of commodity plastics with a larger proportion of heavy pigments. Due to the higher loads of heavy metals, the inclusion of paint resins into MP studies is highly relevant, particularly from an ecotoxicological perspective[44].

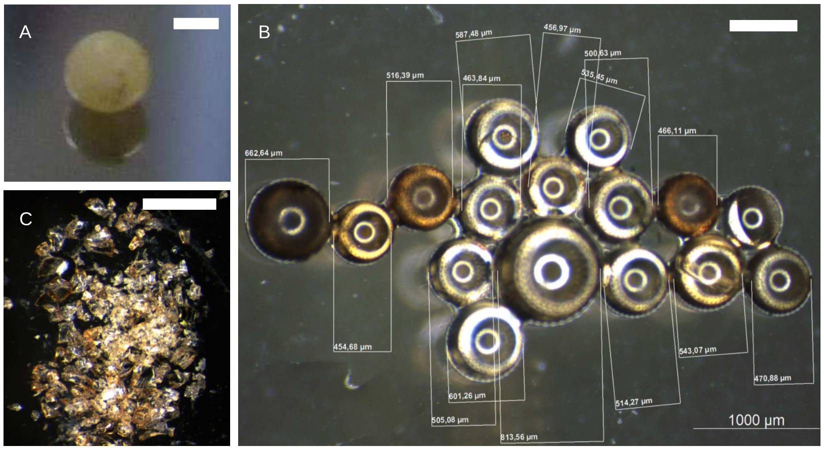

Station	Polymer type	Size [μm]	Colour	Abundance [kg^{-1} DW]	$N_{spectra}$	N_{total}	Fig.
S1	PS	500-1100	opaque, non-transparent	85	8	21	A
S2	SPSS, CPSS	500 - 900	transparent - orange – brown - black	24 ± 21	15	54	B
S5	SPSS, PS, PS-DVB	all above	all above	317	9	214	A, B

Fig. S1. Specifications on the identified group of PS beads, displayed via a selection of images (A-C) and details per station in the table beneath. A - opaque PS bead, B - ion exchanger beads based on sulfonated PS in different variations in size and colour, C - disintegration of ion exchanger bead into particles of $< 50\ \mu m$ caused by the exertion of pressure of the ATR-FTIR crystal. This was likely fostered by physical degradation of ion exchangers for instance by oxidation[48]. Scale bars equal 500 μm.

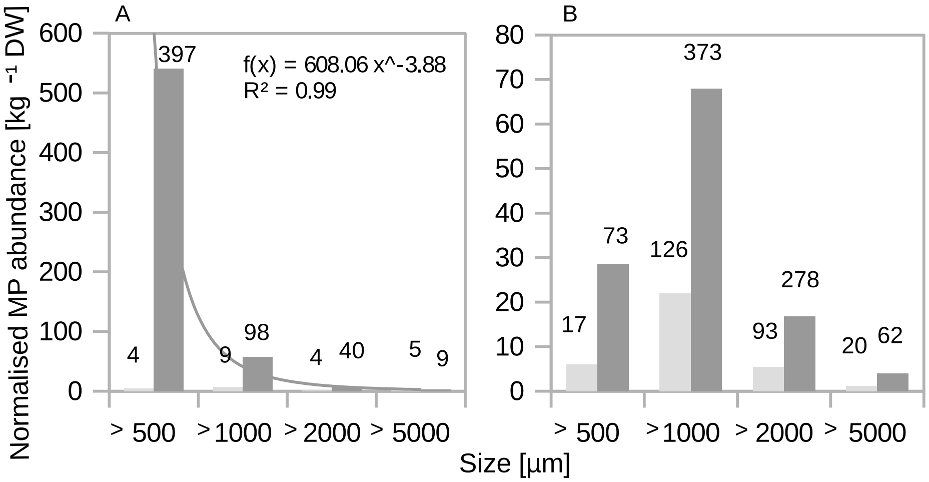

Fig. S2. Normalised size distribution of HD (dark grey) and LD polymers (light grey) in (A) the Warnow estuary and (B) the Alter Strom. Absolute number counts n of measured MP are shown above the bars.

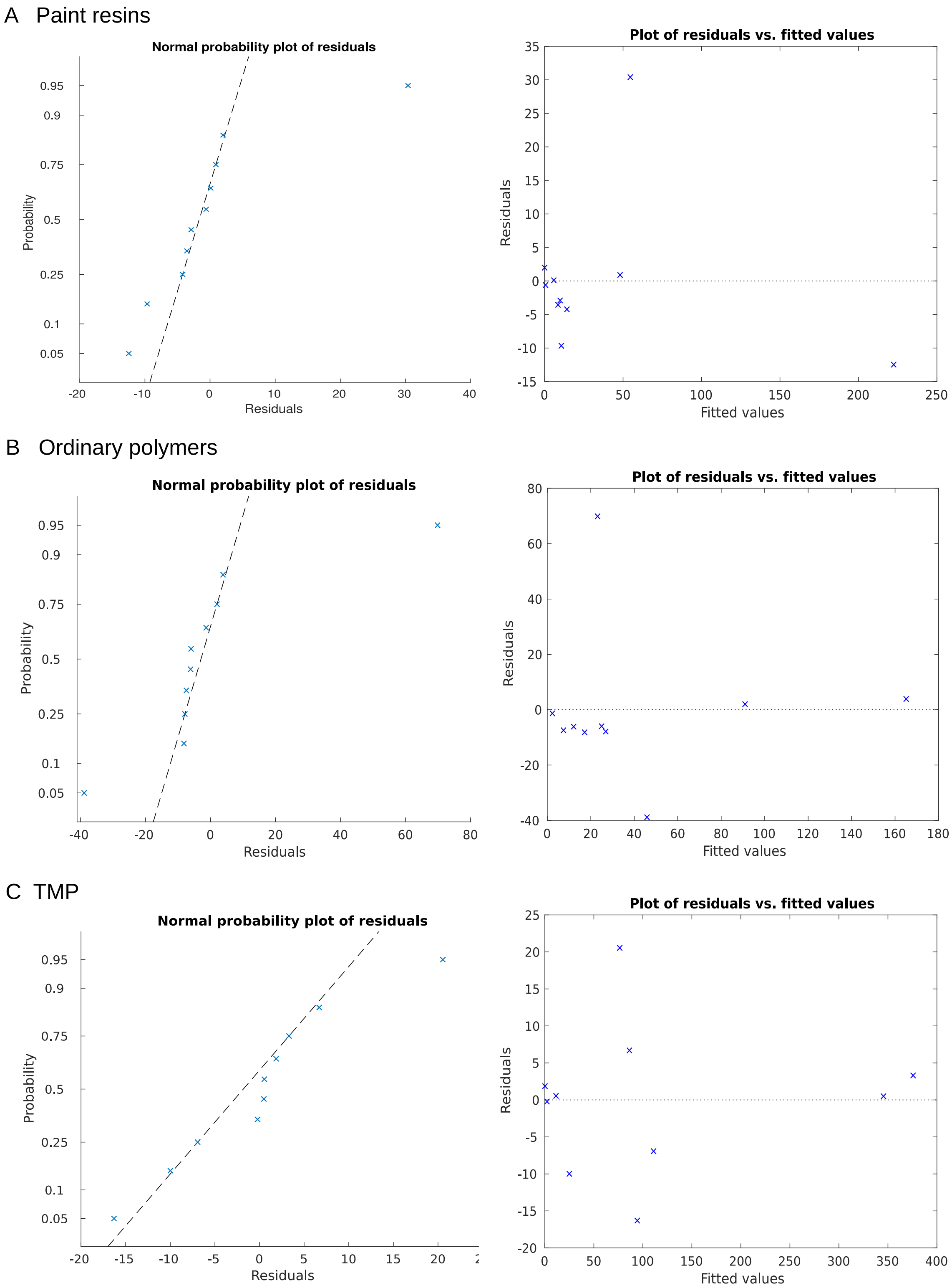

Fig. S3. Plots showing the equality of residual variances and normality of residuals of (A) paint resins, (B) ordinary polymers and (C) TMP. Residual analysis ensured that GLM assumptions of homoscedasticity and normality hold.

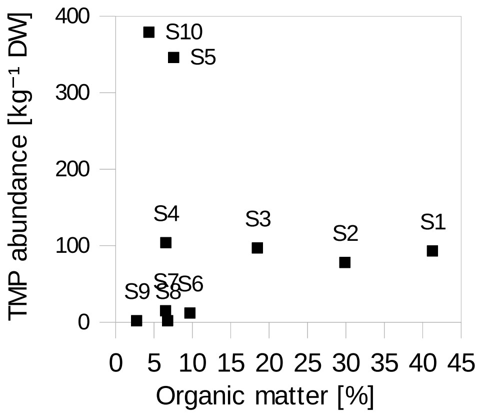

Fig. S4. Organic matter against TMP abundance.

Table 1. Comparison of systemic parameters of MP studies providing data on sediment grain size distribution. For "n.a." no or imprecise data was available. Significant correlations are marked with asterisks. No correlations are marked with minus with the suspected reasons in italic type. More studies provided data showing no or very weak correlations[19–21], however, study design and thus reasons for a low fit were comparable to the two listed. Note that correlation coefficients (r) were calculated based on log transformed MP data, as shown in Fig. 4E.

	**Strand2013**[14]	**Vianello2013**[16]	**Ballent2016**[9]	**present**	**Alomar2016**[17]	**Renzi2018**[18]
**Min. Size [μ m]**	38	32	63	500	63	400
**Location**	Baltic, North Sea, DK	Lagoon of Venice, IT	Lake Ontario, CA	Warnow estuary, DE	Mallorca, ES	Adriatic Sea, IT
**Compartment**	coastal, marine	coastal	limnic	coastal	coastal	coastal, marine
**Polymer analysis**	partly	yes	yes	yes	*no*	*no*
**Max. density [g cm^{-3}]**	1.2	1.2	1.5	1.8	n.a.	1.2
**Number of samples**	22	10	50	10	*6*	*7*
**Sediment analysis technique**	sieve&weigh	sieve&weigh	sieve&weigh	Laser-diffraction	sieve&weigh	sieve&weigh
**Spatial connectivity**	low	high	medium	high	medium	high
**Temporal connectivity**	n.a.	high	low	high	medium	high
**Sediment variation**	high	high	high	high	*low*	*low*
**r**	0.48*	0.86*	0.68*	0.86*	-	-
**p-value**	0.02	0.001	< 0.001	0.001	-	-

Table 2. Specification on sampling stations in the Baltic Sea, both sediment as well as sediment trap samples.

**Sediment samples**	**Date of sampling**	**Latitude**	**Longitude**	**Depth [m]**
Arkona basin	02/09/2015	54.914 N	13.838 E	45
Gotland basin	22/08/2015	57.307 N	20.077 E	248
**Sediment trap samples**				
Arkona basin	12/2012-01/2014	54.884 N	13.862 E	35
Gotland basin	02/2013-11/2013	57.305 N	20.077 E	196

Table 3. Predictor variables, potential MP source terms and environmental parameters, for GLM test including measurements, parameter definition, tools used and references of data acquirement per station (St.).

St.	dist_WWTP [m]	dist_recyc [m]	dist_marina [m]	pop_density _1000m [#]	num_tourism _1000m [#]	dist_rain water[m]	dist_mixed water[m]	num_metal _1000m[#]	num_ind _1000m[#]	OM [%]	TOC/N [%]	$CaCO_3$ [%]	grain size $<63\mu m$[%]	depth [m]	max_shear stress[Nm^{-2}]	SAL [PSU]
S1	5220	2240	1428	10961	75	223	2700	1	0	41.3	14.3	8.7	65.9	1.5	-	1
S2	1980	2440	85	17298	109	97	233	4	0	29.9	14.3	11.8	48.1	2.5	0.047	2
S3	230	2240	501	851	8	203	459	5	6	18.5	14.3	14.8	48.1	1.5	0.043	3
S4	1040	1480	43	478	9	395	1247	7	15	6.5	9.9	12.2	56.4	7.0	0.048	4
S5	2970	480	440	468	6	320	3169	6	3	7.6	9.5	38.0	57.4	1.6	0.094	4
S6	4530	2050	372	9700	33	683	4710	1	0	9.7	11.3	10.3	38.7	1.2	0.042	6
S7	6000	3560	610	1217	4	116	6192	0	3	6.1	10.1	15.3	48.3	6.5	0.036	10
S8	8770	8720	2373	0	0	2372	11500	0	0	6.2	9.0	9.3	29.8	3.5	0.111	12
S9	11180	7340	63	1826	11	301	9400	0	0	0.5	8.2	1.5	7.3	4.0	0.032	12
S10	11180	7000	1	5838	121	150	9400	1	0	10.8	8.8	12.2	95.8	3.5	0.02	11
definition	Distance to the nearest WWTP within the water layer	Distance to the nearest recycling station following water paths	Distance to the nearest point of the nearest marina within the water layer	Cumulative population density of the statistical blocks that are within the 1000m radius of the station	Cumulative number of typical touristic activity points defined by number of restaurants, bars, cafes, beer gardens, fast food restaurants hotels, parks, zoos, picnic sites, viewpoints, museums, tourist information points, guest houses, caravan sites, coffee shops, artworks.	Distance to nearest rain water drainage point, including second order streams	Distance to nearest rain water drainage point, including second order streams	Number of metal industry/ companies within a 1000m radius	Number of industrial sectors within a 1000m radius	TOC=TC-TIC, organic matter (OM) = TOC * 2.22, averaged from two sample replicates	TOC divided by Total Nitrogen, averaged from two sample replicates	Calci-um carbonate	Measured < 1mm, siliclastic sediments	Water depth	maximum shear stress over one year model run (2014)	min. salinity depth averaged, Dec 2005
tools	Measure line, QGIS	Measure line, QGIS	NNJoin plugin (Nearest Neighbor Analysis), Measure line, QGIS	Geoprocessing tool-buffer and Intersection, QGIS	Data management Tools-Merge vector layers, Geoprocessing tool-buffer and Intersection, QGIS	NNJoin plugin, QGIS	NNJoin plugin, Measure line, QGIS	google maps measuring tool, search for 'metal'	Geo processing tool-buffer and Intersection, QGIS	see Geological and chemical analysis				In-situ measured	modelled in matlab	estimate
original data reference	open street maps	Abfall-wirtschaft, Abfall-entsorgungs-anlagen, Recyclinganlagen, www.umweltkarten.mv-regierung.de (2017)	open street maps	www.opendata-hro.de/dataset/statistische bloecke (2017); Population densities from Kommunale Statistikstelle, Rostock	Data from 'Quick OSM' QGIS plugin[24,25], Based on Top3 local tourist activities, Tourismuskonzeption 2022	Coordinates of inlets and max. discharge volume: EuraWasser-Nord GmbH	Coordinates of inlets and max. discharge volume: EuraWasser-Nord GmbH	google maps	Data from 'Quick OSM' QGIS plugin						see section Hydrodynamic model	Das Ostseemodell der BAW Technische Dokumentation, 2015
